# *Mycobacterium lepromatosis* Lepromatous Leprosy in US Citizen Who Traveled to Disease-Endemic Areas

**DOI:** 10.3201/eid2502.171895

**Published:** 2019-02

**Authors:** Gaurav Sharma, Vishnu Dutt Sharma

**Affiliations:** University of Miami, Miami, Florida, USA (G. Sharma);; National Jalma Institute for Leprosy and Other Mycobacterial Diseases, Agra, India (V.D. Sharma)

**Keywords:** *Mycobacterium lepromatosis*, leprosy, travel-related hazard, host susceptibility, United States, bacteria

**To the Editor:** Virk et al. (*1*) reported a *Mycobacterium lepromatosis* infection in a US citizen with a history of multiple international travels and concluded that *M. lepromatosis* lepromatous leprosy is a travel-related hazard for travelers to endemic areas. The conclusions drawn, however, need extensive support of thoroughly conducted case studies before generalizing *M. lepromatosis* as a travel-related hazard. 

In the case report, the exact source of *M. lepromatosis* infection was unclear. Moreover, experimental evidence used in this work are not enough to prove that *M. lepromatosis* is a travel-related hazard. Confirming a source of infection by DNA fingerprinting of *M. lepromatosis* can be ideal to rule out infection from unreported native patients or environmental reservoirs (*2*).

It is possible that the patient in this report may have contracted *M. lepromatosis* infection as a result of his host-susceptible genetic factors. Host genetic susceptibility to leprosy is complicated because of the genetics of *M. lepromatosis*, interaction between genetic and environmental factors, gene–gene interactions, and ethnicity (*3*). Host genetics plays a major role in determining a person’s risk of developing clinical leprosy. Thus, even a short trip to a leprosy-endemic country is sufficient for a host susceptible to *M. lepromatosis* to acquire an infection. The host immune response influences the course of leprosy infection; it is challenging to understand the genetics of disease susceptibility and immunopathogenesis of leprosy (*4**,**5*).

With an inference of only a single case study, it is hard to say that *M. lepromatosis* lepromatous leprosy is a travel-related hazard for all US citizens. More surveillance data, such as patients’ immunity toward the disease, their genetic susceptibility, and travel history, are needed to explore the travel-related hazard. In addition, evolutionary knowledge and how widely the disease is circulating in nonendemic regions will help in understanding the nature of the disease.
